# Fatherhood status in relation to prostate cancer risks in two large U.S.‐based prospective cohort studies

**DOI:** 10.1002/cam4.3606

**Published:** 2020-11-21

**Authors:** Ashley M. Geczik, Scott P. Kelly, Ruth M. Pfeiffer, Wen‐Yi Huang, Linda M. Liao, Cindy K. Zhou, Louise A. Brinton, Michael B. Cook

**Affiliations:** ^1^ Division of Cancer Epidemiology and Genetics National Cancer Institute NIH DHHS Bethesda MD USA

**Keywords:** family size, fertility, offspring, prostate neoplasms, reproductive history, risk factors

## Abstract

**Background:**

Despite the high incidence and mortality of prostate cancer (PCa) in the Unites States, few risk factors have been consistently linked with these PCa outcomes. Assessing proxies of reproductive factors may offer insights into PCa pathogenesis. In this study, we examined fatherhood status as a proxy of fertility in relation to total, nonaggressive, aggressive, and fatal PCa.

**Methods:**

We examined participants of two cohorts, the NIH‐AARP Diet and Health (NIH‐AARP) Study and Prostate, Lung, Colorectal, and Ovarian (PLCO) Cancer Screening Trial. We used Cox proportional hazards regression to estimate hazard ratios (HRs) and 95% confidence intervals of associations between fatherhood status and number of children sired in relation to PCa incidence.

**Results:**

Fatherhood status (one or more children vs. childless) was positively associated with total PCa risk in NIH‐AARP or PLCO, but was not statistically significant (*p* = 0.06 and 0.55, respectively). Number of children sired indicated a slightly elevated risk of total PCa, but HRs were rarely significant and were of a fairly constant magnitude with no discernable trend relative to the childless referent group. Associations were similar for nonaggressive and aggressive PCa. The trend test for fatal PCa was statistically significant in NIH‐AARP (*p*_trend_ < 0.01), despite none of the individual categorical point estimates reaching this threshold.

**Conclusion:**

This study provides tentative evidence that fathering children is associated with a slightly increased PCa risk. Future research should strive to assess better proxies of reproductive function in relation to aggressive and fatal PCa to provide more specific evidence for this putative relationship.

## Graphical abstract

Fatherhood status (one or more children vs. childless) was not associated with prostate cancer risk in NIH‐AARP or PLCO. However, in NIH‐AARP increased number of children sired was associated with an increased total prostate cancer, but this association did not present by disease aggressiveness.

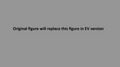

## 1 INTRODUCTION

In the United States (U.S.), the 2020 estimates for the number of new incident prostate cancer (PCa) cases is 191,930 and the estimated number of PCa‐related deaths is 33,330.^1^ Despite PCa being so prevalent, few factors have been consistently associated with PCa risk. Age, family history of PCa, race/ethnicity, geographic location (including migration), and germline genetic variants are among the established risk factors.^2^, ^3^, ^4^ However, other risk factors that have demonstrated putative associations with PCa require further examination. In particular, sex‐steroid hormones and reproductive factors have long been considered to play a role in PCa pathogenesis and progression. The number of children sired (fatherhood status) has been studied as a proxy exposure of fertility.^5^ Despite a systematic review and meta‐analysis of 11 studies reporting a reduced risk of PCa associated with being childless (OR 0.91, 95% CI 0.87–0.96), there was significant between‐study heterogeneity (*p* < 0.001, *I* ^2^ = 88%).^6^ A more recent study within the UK Biobank Cohort did find a similarly reduced PCa risk associated with having never fathered children (HR 0.89, 95% CI 0.81–0.97),^7^ possibly indicating that a greater evidence base may help further elucidate this association as well as the sources of heterogeneity. This is supported by the fact that some prior studies have been limited in their ability to assess these relationships due to imprecise definitions of fatherhood status, high potentials for residual confounding, small sample sizes, and limited assessment of disease aggressiveness.

In an effort to provide more definitive findings, we examined fatherhood status as a proxy for fertility in men in relation to total, nonaggressive, aggressive, and fatal PCa risks in two large U.S.‐based cohort studies; the NIH‐AARP Diet and Health (NIH‐AARP) Study and the Prostate, Lung, Colorectal, and Ovarian (PLCO) Cancer Screening Trial. This study includes longer follow‐up time than previous studies and includes stratification by tumor aggressiveness.

## 2 METHODS

### 2.1 Study population

#### 2.1.1 NIH‐AARP diet and health study

This study and its participants were previously described.^8^ In brief, 567,169 individuals were enrolled from 1995 to 1996 via a baseline questionnaire (BQ), and followed up by a risk factor questionnaire (RFQ) in 1996 to 1997. The RFQ included questions about fatherhood and 334,905 of the BQ respondents returned a valid RFQ questionnaire. From these 334,905 individuals, we excluded women (*n* = 136,407), proxy respondents (*n* = 10,383), death certificate only cases of prostate cancer without a confirmed prostate cancer diagnosis (*n* = 1580), men diagnosed with any cancer (excluding nonmelanoma skin cancer) prior to the return of their RFQ questionnaire (*n* = 9634), men diagnosed with carcinoma in situ or stage 0 PCa (*n* = 62), men with diagnosis dates equal to the date of RFQ questionnaire return (10), and men with incomplete fatherhood responses (missing number of sons and/or number of daughters, *n* = 14,904), resulting in 161,925 men in our analytic population. In a sensitivity analysis, we added back into the analytic population men who had partially incomplete fatherhood responses (missing number of sons or missing number of daughters, with the missing response recoded to “0” providing for a total sensitivity analytic population of 162,894 [Table S1]).

For the RFQ questionnaire the following questions were asked in reference to number of children sired: “Do you have any full‐ or half‐sisters, full‐ or half‐brothers, daughters, or sons, either living or deceased? Include blood relatives only (if no, skip ahead),” “How many daughters do you have, both living and deceased? Include blood relatives only,” and “How many sons do you have, both living and deceased? Include blood relatives only.”

#### 2.1.2 PLCO cancer screening trial

This study and its participants were previously described.^9^ In brief, 154,887 individuals were enrolled from 1993 to 2001. A baseline questionnaire (BQ) was completed at enrollment and a supplemental questionnaire (SQX) that included questions on fatherhood was sent to all participants during 2006–2008. 102,884 of the BQ respondents returned a valid SQX questionnaire. From these 102,884 individuals, we excluded women (*n* = 54,679), men with any cancer diagnosis (excluding nonmelanoma skin cancer) prior to the return of their SQX questionnaire (*n* = 8105), and incomplete fatherhood responses (*n* = 13,252), resulting in 26,848 men in our analytic population. The sensitivity analysis with recoded partially incomplete fatherhood responses (as done for NIH‐AARP) added back 3519 men to the PLCO cohort, providing for a total sensitivity analytic population of 30,367 (Table S1).

For the SQX questionnaire, there were check boxes for respondents to record numbers of isisters, brothers, daughters, and sons. The question that headed these response check boxes were as follows: “How many of each of the following blood relatives (do not count half‐sisters or half‐brothers) do/did you have? (Please include any deceased).”

### 2.2 Identification of incident PCa cases and PCa‐specific mortality

The primary outcome of interest for our analyses was first incident PCa, as well as the subsets of nonaggressive and aggressive PCa. We defined nonaggressive and aggressive PCa using the clinical cancer stage and Gleason score information from both the NIH‐AARP and PLCO cohorts. Clinical cancer stage was determined using the TNM staging system and categorized according to the American Joint Committee on Cancer (AJCC) Staging Manual, fifth edition. For our analysis, nonaggressive PCa was defined as clinical cancer stage ≤II or Gleason score <8, and aggressive PCa was defined as clinical cancer stage ≥III, Gleason score ≥8 and/or fatal PCa for NIH‐AARP and PLCO. The nonaggressive and aggressive PCa cases do not sum to total PCa, since some individuals with confirmed PCa did not report data on stage or grade.

Our secondary outcome of interest was fatal PCa, which was defined as PCa being the underlying cause of death with a prior confirmed PCa diagnosis. Fatal PCa cases were ascertained through periodic linkage to the National Death Index (NDI), cancer registry linkage, and Social Security Administration Death Master File, with confirmation from proxy responses.^8^ Due to limited fatal PCa cases in the PLCO cohort (*n* = 3 childless men, *n* = 69 fathers), we were only able to examine this outcome in the NIH‐AARP cohort.

### 2.3 Statistical analyses

Cox proportional hazards regression models were used to estimate hazard ratios (HRs) and 95% confidence intervals (CIs) for the associations between fatherhood status and PCa incidence and mortality. Tests for trend were conducted by treating the number of offspring as a continuous variable in the Cox regression model. For our analyses of incident PCa risks, follow‐up time started at calendar month (from which offspring status was recalled) and continued until event of interest (incident PCa) or right censoring due to loss to follow‐up, death, incidence of other cancer diagnosis, or end of follow‐up (NIH‐AARP: 31 December 2011; PLCO: 31 December 2014), whichever occurred first. For our analysis of fatal PCa, follow‐up time started at calendar month (from which offspring status was recalled) and continued until event of interest (PCa‐specific mortality) or right censoring due to loss to follow‐up, death due to another cause, or end of follow‐up up (NIH‐AARP: 31 December 2011; PLCO: 31 December 2014). Calendar time was used as the underlying time metric and the baseline hazards were stratified by categorical age groups (NIH‐AARP: ≤55, 55–60, 60–65, 65–70, and >70 years old; PLCO: ≤65, 65–70, 70–75, 75–80, and >80 years old). We assessed the proportional hazards assumption by testing whether smoothed, scaled Schoenfeld residuals significantly deviated from a nonzero slope when regressed against log time.

Covariates that were, a priori, deemed to be potential confounders included: education, race, marital status, state/center, and randomization arm (PLCO). All statistical tests were two‐sided and statistical significance was defined as *p* < 0.05.

To assess potential effect measure modification of marital status and PSA screening history, we compared nested models with the inclusion of multiplicative interaction terms and tested whether model fit was significantly improved using the likelihood ratio test. Stratified analyses were conducted if statistically significant (*p* < 0.05) effect measure modification was detected.

Sensitivity analyses included: (a) recoding partially incomplete fatherhood responses (daughter or son) to zero; (b) reclassifying marital status from three categories (never married, formerly married/widowed, and currently married) to four (never married, married, formerly married, and widowed); and, (c) excluding never married men.^8^ Statistical analyses were conducted using SAS version 9.4 (SAS Institute Inc.).

## 3 RESULTS

Descriptive statistics for each of the two cohorts are shown in Table 1. At the time that fatherhood status was ascertained, men in the NIH‐AARP cohort had a younger average age (63 ± 5 years) compared with men in PLCO (71 ± 6 years). Men enrolled in NIH‐AARP were more likely to have obtained a college or higher degree (70%) compared with men enrolled in PLCO (46%). Higher proportions of NIH‐AARP (72% prostate‐specific antigen [PSA], 84% digital rectal examination [DRE]) participants had undergone prostate cancer screening compared with PLCO trial (47% PSA, 57% DRE) participants. Annual household income, provided via census collected data for NIH‐AARP participants, showed that NIH‐AARP and PLCO had a similar distribution with a majority of men falling in the $20,000–$49,999 income bracket followed by the $50,000–$99,999 income bracket. Most men reported no family history of prostate cancer in both NIH‐AARP (86%) and PLCO (91%). Other descriptive characteristics were similar across both cohorts.

**Table 1:** Descriptive characteristics of men by cohort according to prostate cancer status

	NIH‐AARP										PLCO									
	Total population		Prostate cancer cases		Nonaggressive prostate cancer		Aggressive prostate cancer		Fatal prostate cancer		Total population		Prostate cancer cases		Nonaggressive prostate cancer		Aggressive prostate cancer		Fatal prostate cancer	
Descriptive characteristics	*N*	%	*N*	%	*N*	%	*N*	%	*N*	%	*N*	%	*N*	%	*N*	%	*N*	%	*N*	%
Number of participants	161,925		17,841		13,041		4512		710		26,848		1779		1399		377		72	
Enty age (mean, SD)	63.0 ± 5.3		63.6 ± 5.0		63.6 ± 5.0		63.6 ± 5.0		65.2 ± 4.4		71.2 ± 5.8		70.6 ± 5.4		70.1 ± 5.2		72.5 ± 5.7		75.0 ± 6.1	
BMI (mean, SD)	27.1 ± 4.2		26.8 ± 3.8		27.0 ± 3.8		27.0 ± 4.0		27.2 ± 4.0		27.7 ± 4.4		27.5 ± 4.1		27.5 ± 4.0		27.5 ± 4.4		27.0 ± 4.0	
Height (mean, SD)	70.2 ± 2.9		70.2 ± 2.9		70.2 ± 2.9		70.3 ± 2.9		70.5 ± 2.9		69.8 ± 2.8		69.8 ± 2.8		69.9 ± 2.8		69.5 ± 2.8		69.0 ± 2.7	
Fatherhood status																				
Childless	17,986	11.1	1794	10.1	1315	10.1	449	10.0	77	10.8	1922	7.2	123	6.9	102	7.3	21	5.6	3	4.2
One or more children	143,939	88.9	16,047	89.9	11,726	89.9	4063	90.0	633	89.2	24,926	92.8	1656	93.1	1297	92.7	356	94.4	69	95.8
Marital status																				
Currently married/living as married	138,431	85.5	15,548	87.1	11,404	87.4	138	3.1	27	3.8	22,441	83.6	1526	85.8	1211	86.6	312	82.8	54	75.0
Formerly married/widowed	17,059	10.5	1682	9.4	1197	9.2	453	10.0	93	13.1	3735	13.9	207	11.6	148	10.6	59	15.6	17	23.6
Never married	5613	3.5	536	3.0	386	3.0	3903	86.5	586	82.5	485	1.8	31	1.7	27	1.9	4	1.1	1	1.4
Unknown	822	0.5	75	0.4	54	0.4	18	0.4	4	0.6	187	0.7	15	0.8	13	0.9	2	0.5	0	0.0
Education																				
<12 years	7399	4.6	726	4.1	521	4.0	179	4.0	31	4.4	1440	5.4	71	4.0	54	3.9	17	4.5	4	5.6
High school/some college	38,051	23.5	3930	22.0	2868	22.0	1012	22.4	172	24.2	12,939	48.2	838	47.1	646	46.2	191	50.7	39	54.2
College degree or higher	112,898	69.7	12,806	71.8	9370	71.9	3231	71.6	489	68.9	12,414	46.2	865	48.6	695	49.7	168	44.6	29	40.3
Unknown	3577	2.2	379	2.1	282	2.2	90	2.0	18	2.5	55	0.2	5	0.3	4	0.3	1	0.3	0	0.0
Race																				
White	151,812	93.8	16,580	92.9	12,114	92.9	4193	92.9	662	93.2	24,307	90.5	1584	89.0	1250	89.3	331	87.8	61	84.7
Black	3514	2.2	621	3.5	459	3.5	156	3.5	29	4.1	516	1.9	53	3.0	36	2.6	17	4.5	3	4.2
Other	4982	3.1	470	2.6	335	2.6	128	2.8	10	1.4	1494	5.6	108	6.1	84	6.0	24	6.4	6	8.3
Unknown	1617	1.0	170	1.0	133	1.0	35	0.8	9	1.3	531	2.0	34	1.9	29	2.1	5	1.3	2	2.8
Income																				
<$20,000	1620	1.0	166	0.9	113	0.9	50	1.1	10	1.4	1913	7.1	103	5.8	76	5.4	26	6.9	9	12.5
$20,000–$49,999	77,394	47.8	8234	46.2	6033	46.3	2051	45.5	343	48.3	9817	36.6	600	33.7	448	32.0	152	40.3	31	43.1
$50,000–$99,999	73,394	45.3	8269	46.3	6044	46.3	2105	46.7	321	45.2	8429	31.4	594	33.4	501	35.8	92	24.4	14	19.4
$100,000–$200,000	9419	5.8	1162	6.5	842	6.5	306	6.8	36	5.1	2627	9.8	226	12.7	187	13.4	39	10.3	4	5.6
>$200,000	98	0.1	10	0.1	9	0.1	0	0.0	0	0.0	480	1.8	42	2.4	33	2.4	9	2.4	1	1.4
Prefer not to answer											2595	9.7	144	8.1	97	6.9	46	12.2	10	13.9
Unknown											987	3.7	70	3.9	57	4.1	13	3.4	3	4.2
Smoking status																				
Never	48,140	29.7	5994	33.6	4418	33.9	1484	32.9	176	24.8	9768	36.4	697	39.2	555	39.7	141	37.4	27	37.5
Former	93,296	57.6	9957	55.8	7299	56.0	2499	55.4	412	58.0	15,037	56.0	969	54.5	746	53.3	211	56.0	40	55.6
Current	14,997	9.3	1309	7.3	914	7.0	373	8.3	94	13.2	1776	6.6	106	6.0	87	6.2	19	5.0	4	5.6
Unknown	5492	3.4	581	3.3	410	3.1	156	3.5	28	3.9	267	1.0	17	1.0	11	0.8	6	1.6	1	1.4
Family first degree history of prostate cancer																				
No	139,468	86.1	14,997	84.1	10,930	83.8	3817	84.6	575	81.0	24,304	90.5	1570	88.3	1232	88.1	335	88.9	66	91.7
Yes	4980	3.1	949	5.3	707	5.4	226	5.0	45	6.3	1943	7.2	187	10.5	151	10.8	36	9.5	6	8.3
Unknown	17,477	10.8	1895	10.6	1404	10.8	469	10.4	90	12.7	601	2.2	22	1.2	16	1.1	6	1.6	0	0.0
PSA screening history																				
No	33,290	20.6	3098	17.4	2100	16.1	942	20.9	197	27.7	12,034	44.8	801	45.0	626	44.7	174	46.2	37	51.4
Yes	116,118	71.7	13,568	76.0	10,116	77.6	3231	71.6	455	64.1	12,604	46.9	832	46.8	663	47.4	167	44.3	29	40.3
Unknown	12,517	7.7	1175	6.6	825	6.3	339	7.5	58	8.2	2210	8.2	146	8.2	110	7.9	36	9.5	6	8.3
Digital rectal examination history																				
No	22,273	13.8	2090	11.7	1411	10.8	639	14.2	139	19.6	10,753	40.1	726	40.8	571	40.8	154	40.8	30	41.7
Yes	135,619	83.8	15,351	86.0	11,348	87.0	3763	83.4	555	78.2	15,418	57.4	1013	56.9	798	57.0	213	56.5	41	56.9
Unknown	4033	2.5	400	2.2	282	2.2	110	2.4	16	2.3	677	2.5	40	2.2	30	2.1	10	2.7	1	1.4
Diabetes																				
No	146,449	90.4	16,697	93.6	12,221	93.7	4209	93.3	660	93.0	21,831	81.3	1479	83.1	1177	84.1	300	79.6	55	76.4
Yes	15,476	9.6	1144	6.4	820	6.3	303	6.7	50	7.0	4237	15.8	246	13.8	180	12.9	65	17.2	15	20.8
Unknown	N/A^a^	N/A^a^	N/A^a^	N/A^a^	N/A^a^	N/A^a^	N/A^a^	N/A^a^	N/A^a^	N/A^a^	780	2.9	54	3.0	42	3.0	12	3.2	2	2.8

Abbreviations: BMI, body mass index; PSA, prostate specific antigen.

^a^ Not applicable because the question only elicited affirmative responses for this condition and was therefore coded binary.

The median person‐years of follow‐up for NIH‐AARP was 15.1 and for PLCO was 7.9. There were 17,841 incident PCas in NIH‐AARP and 1779 incident PCas in PLCO. Of these, 4512 (25%) and 377 (21%) were aggressive in NIH‐AARP and PLCO, respectively (Table 1). There were 710 fatal PCas in NIH‐AARP and 72 in PLCO (Table 1). When we included individuals that had partially incomplete fatherhood responses, there were very small increases in these numbers (total incident PCas = 17,941 in NIH‐AARP and 1987 in PLCO; Table S1).

For NIH‐AARP, the association of fatherhood status (one or more child vs. childless) with total PCa was positive (HR=1.06), but did not reach the designated threshold for statistical significance (*p* = 0.06). Associations between fatherhood status and nonaggressive, aggressive, and fatal PCa risks were in a similar direction, but with larger *p* values (Table 2). For PLCO, associations were also positive, but not statistically significant for total, nonaggressive, and aggressive PCa risk. The 72 fatal PCa cases in PLCO were not amenable to a converged model likely due to the fact that only three men had indicated they were childless. Associations were similar when including the small additional number of men with partially incomplete fatherhood questionnaire responses (Table S2).

**Table 2:** Adjusted hazard ratios (HRs) and 95% confidence intervals (CI) for each cohort according to prostate cancer status with baseline hazards stratified by age group

	NIH‐AARP^a^											
	Total cases (*n* = 17,841)			Nonaggressive cases (*n* = 13,041)			Aggressive cases (*n* = 4512)			Fatal cases (*n* = 710)		
	Cases	HR (95% CI)	*p* value	Cases	HR (95% CI)	*p* value	Cases	HR (95% CI)	*p* value	Cases	HR (95% CI)	*p* value
Fatherhood status												
Childless	1794	Reference		1315	Reference		449	Reference		77	Reference	
1 or more children	16,047	1.06 (1.00, 1.12)	0.06	11,726	1.04 (0.98–1.11)	0.22	4063	1.09 (0.97–1.22)	0.13	633	1.04 (0.78–1.39)	0.78

	PLCO^b^											
	Total cases (*n* = 1779)			Nonaggressive cases (*n* = 1399)			Aggressive cases (*n* = 377)			Fatal cases (*n* = 72)^c^		
	Cases	HR (95% CI)	*p* value	Cases	HR (95% CI)	*p* value	Cases	HR (95% CI)	*p* value	Cases	HR (95% CI)	*p* value
Fatherhood status												
Childless	123	Reference		102	Reference		21	Reference		3	Reference	—
1 or more children	1656	1.07 (0.87–1.32)	0.55	1297	1.03 (0.81–1.29)	0.83	356	1.24 (0.76–2.01)	0.39	69	—	—

^a^ Adjusted for: education status (<12 years, HS/some college, college graduate or higher, unknown), race (White/non‐Hispanic, Black/non‐Hispanic, other, unknown), marital status (never married, formerly married/widowed, currently married, unknown), and state of residence at baseline (California, Florida, Georgia, Louisiana, Michigan, North Carolina, New Jersey, Pennsylvania).

^b^ Adjusted for: education status (<12 years, HS/some college, college graduate or higher, unknown), race (White/non‐Hispanic, Black/non‐Hispanic, other, unknown), marital status (never married, formerly married/widowed, currently married, unknown), randomization arm (intervention, control), and center of enrollment (University of Colorado, Georgetown University, Pacific Health Research and Education Institute (Honolulu), Henry Ford Health System, University of Minnesota, Washington University in St. Louis, University of Pittsburgh, University of Utah, Marshfield Clinic Research Foundation, University of Alabama at Birmingham).

^c^ PLCO prostate cancer specific mortality was not able to be examined for survival analysis to estimate hazard ratios due to limited number of childless men having prostate cancer.

Assessment of the association between number of children sired and PCa risk were similar across the two cohorts (Table 3), with slightly elevated HRs that were rarely significant and were of a fairly constant magnitude with no discernable trend relative to the childless referent group. This was true for total, nonaggressive, and aggressive PCa, with the latter showing a slightly stronger, albeit nonsignificant, association in PLCO compared with NIH‐AARP. The trend test for fatal PCa was statistically significant in NIH‐AARP (*p*_trend_ = 0.0047), despite none of the individual categorical point estimates reaching this threshold.

**Table 3:** Adjusted hazard ratios (HRs) and 95% confidence intervals (CI) for incident prostate cancer cases among fathers for each cohort with baseline hazards stratified by age group.

	NIH‐AARP^a^											
	Total (*n* = 17,841)			Nonaggressive (*n* = 13,041)			Aggressive (*n* = 4512)			Fatal (*n* = 710)		
	Cases	HR (95% CI)	*p* value	Cases	HR (95% CI)	*p* value	Cases	HR (95% CI)	*p* value	Cases	HR (95% CI)	*p* value
Fatherhood status												
Childless	1794	Reference	—	1315	Reference	—	449	Reference	—	77	Reference	—
1 or more children	16,047	1.06 (1.00, 1.12)	0.06	11,726	1.04 (0.98, 1.11)	0.22	4063	1.09 (0.97, 1.22)	0.13	633	1.04 (0.78, 1.39)	0.78
2 or more children	14,392	1.06 (1.00, 1.12)	0.05	10,535	1.05 (0.98, 1.12)	0.18	3634	1.09 (0.97, 1.22)	0.14	572	1.05 (0.79, 1.40)	0.74
3 or more children	8915	1.07 (1.01, 1.13)	0.02	6509	1.05 (0.98, 1.13)	0.13	2256	1.11 (0.98, 1.24)	0.09	381	1.11 (0.83, 1.48)	0.50
4 or more children	4232	1.06 (0.99, 1.13)	0.08	3075	1.04 (0.96, 1.12)	0.33	1086	1.11 (0.98, 1.26)	0.10	209	1.24 (0.91, 1.68)	0.17
5 or more children	1744	1.05 (0.98, 1.13)	0.16	1276	1.04 (0.96, 1.13)	0.37	440	1.09 (0.94, 1.26)	0.26	92	1.29 (0.92, 1.83)	0.14
Trend		1.01 (1.00–1.02)	0.18		1.00 (0.99–1.02)	0.47		1.01 (0.99–1.03)	0.20		1.07 (1.02–1.12)	<0.01

	PLCO^b^											
	Total (*n* = 1779)			Nonaggressive (*n* = 1399)			Aggressive (*n* = 377)			Fatal (*n* = 72)^c^		
	Cases	HR (95% CI)	*p* value	Cases	HR (95% CI)	*p* value	Cases	HR (95% CI)	*p* value	Cases	HR (95% CI)	*p* value
Fatherhood status												
Childless	123	Reference	—	102	Reference	—	21	Reference	—	3	Reference	—
1 or more children	1656	1.07 (0.87, 1.32)	0.55	1297	1.03 (0.81, 1.29)	0.83	356	1.24 (0.76, 2.01)	0.39	69	—	—
2 or more children	1567	1.07 (0.87, 1.33)	0.50	1221	1.03 (0.81, 1.30)	0.82	343	1.27 (0.78, 2.07)	0.34	68	—	—
3 or more children	1080	1.07 (0.86, 1.32)	0.54	832	1.02 (0.81, 1.30)	0.86	246	1.27 (0.77, 2.07)	0.35	53	—	—
4 or more children	609	1.11 (0.89, 1.38)	0.37	470	1.08 (0.84, 1.38)	0.55	137	1.22 (0.73, 2.02)	0.44	33	—	—
5 or more children	298	1.12 (0.89, 1.42)	0.33	230	1.11 (0.85, 1.45)	0.44	67	1.20 (0.70, 2.05)	0.51	13	—	—
Trend		1.01 (0.98–1.04)	0.49		1.01 (0.98–1.04)	0.62		1.02 (0.95–1.08)	0.62		—	—

^a^ Adjusted for: education status (<12 years, HS/some college, college graduate or higher, unknown), race (White/non‐Hispanic, Black/non‐Hispanic, other, unknown), marital status (never married, formerly married/widowed, currently married, unknown), and state of residence at baseline (California, Florida, Georgia, Louisiana, Michigan, North Carolina, New Jersey, Pennsylvania).

^b^ Adjusted for: education status (<12 years, HS/some college, college graduate or higher, unknown), race (White/non‐Hispanic, Black/non‐Hispanic, other, unknown), marital status (never married, formerly married/widowed, currently married, unknown), randomization arm (intervention, control), and center of enrollment (University of Colorado, Georgetown University, Pacific Health Research and Education Institute (Honolulu), Henry Ford Health System, University of Minnesota, Washington University in St. Louis, University of Pittsburgh, University of Utah, Marshfield Clinic Research Foundation, University of Alabama at Birmingham).

^c^ PLCO prostate cancer specific mortality was not able to be examined for survival analysis to estimate hazard ratios due to no childless men having prostate cancer.

There was no evidence of effect measure modification by marital status (total PCa, *p* = 0.23 for NIH‐AARP and *p* = 0.49 for PLCO; nonaggressive PCa, *p* = 0.10 for NIH‐AARP and *p* = 0.51 for PLCO; aggressive PCa, *p* = 0.71 for NIH‐AARP and *p* = 0.88 for PLCO; and fatal PCa, *p* = 0.84 for NIH‐AARP). There was tentative evidence for effect measure modification by PSA screening history in NIH‐AARP (total PCa, *p* = 0.05 for NIH‐AARP and *p* = 0.06 for PLCO; nonaggressive PCA, *p* = 0.37 for NIH‐AARP and *p* = 0.06 for PLCO; aggressive PCa, *p* = 0.04 for NIH‐AARP and *p* = 0.75 for PLCO; and fatal PCa, *p* = 0.99 for NIH‐AARP) and stratified analyses by PSA screening history for NIH‐AARP examining total PCa and aggressive PCa risk (Table S4). Sensitivity analyses of reclassification of marital status and exclusion of never married men did not materially change the presented results (results not shown). Associations for both NIH‐AARP and PLCO cohorts were unaltered when we included subjects with partially incomplete fatherhood responses (Tables S1–S3).

## 4 DISCUSSION

In this study of two large U.S.‐based prospective cohort studies with long follow‐up, we examined the association between fatherhood status and PCa risks. Fatherhood status (one or more children vs. childless) was positively associated with total PCa risk in NIH‐AARP or PLCO, but was not statistically significant. We observed slightly elevated risk of total, nonaggressive, and aggressive PCa risk in relation to number of children sired, but HRs were rarely significant and were of a fairly constant magnitude with no discernable trend relative to the childless referent group. The trend test for fatal PCa, in NIH‐AARP, was statistically significant even though none of the individual categorical point estimates reached this threshold. Overall, this study provides tentative evidence that fathering children is associated with a slightly increased PCa risk.

A prior meta‐analysis of 11 studies has also reported a significant reduction in PCa risk for childless men compared with men who had fathered at least one child (OR 0.91, 95% CI 0.87–0.96), but there was significant between‐study heterogeneity,^6^ underscoring the inconsistency in the existing literature. Previous studies have observed a reduced risk of PCa among childless men compared to men that are fathers.^6^, ^7^ Potential explanations for inconsistent results include the inability to discern intention versus infertility, as well as geographical, financial, and birth cohort differences that could affect the magnitude of such bias. Given the complex wording of questions and responder‐determined skip patterns in extensive questionnaires, exposure ascertainment bias may also contribute to between‐study heterogeneity. Despite these inconsistencies, there is lateral supporting evidence that men whose female partners used in vitro fertilization (IVF) or intracytoplasmic sperm injection (ICSI) had an increased risk of PCa, particularly earlier disease, compared with men who succeeded in natural conception.^10^

Biological explanations that may underlie the observed association between subfertility and PCa may be PSA dynamics,^11^ local (prostate) inflammation,^12^ Y chromosome genetics/epigenetics,^13^ shared genetic etiology such as DNA repair,^14^ environmental exposures such as endocrine disrupting chemicals,^15^ among other hypotheses.^16^, ^17^

Strengths of our study include the similarity of the questionnaires and descriptive characteristics of NIH‐AARP and PLCO for comparison, the large sample sizes available for analysis, and the availability of a range of covariates that we assessed as potential confounders and effect modifiers. Another strength of our study is the use of extended follow‐up data on case ascertainment and mortality for the men in NIH‐AARP from the prior analysis using this cohort (17,841 PCa cases in our analysis vs. 8134 PCa cases^8^ in a prior analysis). A limitation of our study was the small number of childless prostate cancer cases in the PLCO cohort. As such, we recommend a cautious interpretation of the PLCO results and a greater reliance being placed on estimates from NIH‐AARP. The use of childless status as a proxy for subfertility is a limitation and we did not have information to assess to what degree this population represented subfertility/infertility or was attributable to personal preference. Additionally, the high number of men that did not provide responses on number of offspring is a limitation for this study. In examining the questionnaires from NIH‐AARP and PLCO, it is possible that the men accidentally skipped over these questions if they did not have siblings without realizing the questions were relevant to siblings and offspring. A final limitation is that we were unable to examine fatal PCa in the PLCO cohort due to a limited number of outcomes (*n* = 72).

Future research should strive to assess more specific metrics of reproductive function—such as subfertility/infertility, primary/secondary hypogonadism, and hormone therapies—in relation to aggressive and fatal PCa to provide stronger evidence and further insight into these putative relationships.

## CONFLICT OF INTEREST

There were no conflicts of interest.

## AUTHOR CONTRIBUTIONS

Ashley M. Geczik: Study design, statistical analysis, data interpretation, writing, and critical review of the article. Michael B. Cook, Scott P. Kelly, and Louise A. Brinton: Study conception, study design, data interpretation, and critical review of the article. Cindy K. Zhou and Ruth M. Pfeiffer: Study design, statistical analysis consultation, data interpretation, and critical review of the article. Wen‐Yi Huang and Linda M. Liao: Study management, data interpretation, and critical review of the article.

## Supporting information

Supplementary Material

## Data Availability

NIH‐AARP: Data are available upon submitting a proposal to be approved by the NIH‐AARP Steering Committee at https://www.nihaarpstars.com/. PLCO: Requests for access to the data underlying this article should be submitted through the PLCO Cancer Data Access System at https://cdas.cancer.gov/learn/plco/instructions/?type=data.
